# Association of Skilled Nursing Facility Ownership by Health Care Networks With Utilization and Spending

**DOI:** 10.1001/jamanetworkopen.2023.0140

**Published:** 2023-02-20

**Authors:** Stanley Kalata, Ryan Howard, Adrian Diaz, Usha Nuliyahu, Andrew M. Ibrahim, Hari Nathan

**Affiliations:** 1 Department of Surgery, University of Michigan, Ann Arbor; 2 Center for Healthcare Outcomes and Policy, University of Michigan, Ann Arbor; 3 Department of Surgery, The Ohio State University, Columbus

## Abstract

**Importance:**

Health care mergers and acquisitions have increased vertical integration of skilled nursing facilities (SNFs) in health care networks. While vertical integration may result in improved care coordination and quality, it may also lead to excess utilization, as SNFs are paid a per diem rate.

**Objective:**

To determine the association of vertical integration of SNFs within hospital networks with SNF utilization, readmissions, and spending for Medicare beneficiaries undergoing elective hip replacement.

**Design, Setting, and Participants:**

This cross-sectional study evaluated 100% Medicare administrative claims for nonfederal acute care hospitals performing at least 10 elective hip replacements during the study period. Fee-for-service Medicare beneficiaries aged 66 to 99 years who underwent elective hip replacement between January 1, 2016, and December 31, 2017, with continuous Medicare coverage for 3 months before and 6 months after surgery were included. Data were analyzed from February 2 to August 8, 2022.

**Exposures:**

Treatment at a hospital within a network that also owns at least 1 SNF based on the 2017 American Hospital Association survey.

**Main Outcomes and Measures:**

Rates of SNF utilization, 30-day readmissions, and price-standardized 30-day episode payments. Hierarchical multivariable logistic and linear regression clustered at hospitals was performed with adjusting for patient, hospital, and network characteristics.

**Results:**

A total of 150 788 patients (61.4% women; mean [SD] age, 74.3 [6.4] years) underwent hip replacement. After risk adjustment, vertical SNF integration was associated with a higher rate of SNF utilization (21.7% [95% CI, 20.4%-23.0%] vs 19.7% [95% CI, 18.7%-20.7%]; adjusted odds ratio [aOR], 1.15 [95% CI, 1.03-1.29]; *P* = .01) and lower 30-day readmission rate (5.6% [95% CI, 5.4%-5.8%] vs 5.9% [95% CI, 5.7%-6.1%]; aOR, 0.94 [95% CI, 0.89-0.99]; *P* = .03). Despite higher SNF utilization, the total adjusted 30-day episode payments were slightly lower ($20 230 [95% CI, $20 035-$20 425] vs $20 487 [95% CI, $20 314-$20 660]; difference, −$275 [95% CI, −$15 to −$498]; *P* = .04) driven by lower postacute payments and shorter SNF length of stays. Adjusted readmission rates were particularly low for patients not sent to an SNF (3.6% [95% CI, 3.4%-3.7%]; *P* < .001) but were significantly higher for patients with an SNF length of stay less than 5 days (41.3% [95% CI, 39.2%-43.3%]; *P* < .001).

**Conclusions and Relevance:**

In this cross-sectional study of Medicare beneficiaries undergoing elective hip replacements, vertical integration of SNFs in a hospital network was associated with higher rates of SNF utilization and lower rates of readmissions without evidence of higher overall episode payments. These findings support the purported value of integrating SNFs into hospital networks but also suggest that there is room for improving the postoperative care of patients in SNFs early in their stay.

## Key Points

**Question:**

Do Medicare beneficiaries undergoing elective hip replacements in hospital networks that own skilled nursing facilities (SNFs) have differences in SNF utilization, readmissions, or payments compared with those treated at hospitals without vertically integrated SNFs?

**Findings:**

This cross-sectional study of 150 788 patients found that vertical integration of SNFs in a hospital network was associated with higher adjusted rates of overall SNF utilization and lower overall adjusted rates of readmission with similar overall episode payments.

**Meaning:**

These findings suggest that vertical integration of SNFs in a hospital network was associated with modest increases in SNF utilization without overall adversely affecting outcomes or costs.

## Teaser

This cross-sectional study assesses differences in surgical outcomes and spending for Medicare beneficiaries undergoing elective hip replacement procedures in hospital networks that have vertical integration of skilled nursing facilities vs those that do not.

## Introduction

Hospital mergers and acquisitions show no sign of slowing, as over 70% of hospitals today are part of larger health networks, which is twice as many as a decade ago.^1,2^ Financial pressures associated with moving from fee-for-service reimbursement toward bundled payments and population-based payment models, as well as evolving referral patterns, have all been implicated.^3,4^ The shifting of financial risks has also motivated integration of hospitals with post–acute care facilities,^5,6,7,8^ which can include policy-inspired formal arrangements, preferred provider networks, or vertical integration. In 2015, 80% of hospitals owned at least 1 post–acute care service, with 33% owning a skilled nursing facility (SNF).^9^ Vertical integration of SNFs will continue to play a growing role as health care networks strategically acquire these facilities and hospitals gain exposure to these facilities upon joining a network.^7,10^

The impact of vertical integration of SNFs on outcomes, utilization, and costs remains unclear. Purported benefits include improved value from integration, collaboration, and standardization of patient care, improving efficiency of administration and clinical services, and achieving economies of scale.^10,11,12^ Prior work^9,13,14^ has shown decreased costs at hospital-based SNFs compared with freestanding SNFs due to shorter lengths of stay. In addition, vertical integration of SNFs has been shown to decrease readmissions for medical cohorts.^15,16^ However, networks that own SNFs may have a perverse financial incentive to overutilize SNFs. One study of patients undergoing emergency surgery^17^ found that hospital ownership of SNFs was associated with higher rates of nonhome discharge, which included SNFs.

We sought to assess differences in surgical outcomes and spending for Medicare beneficiaries undergoing elective hip replacements in hospitals within networks that had vertical integration of SNFs vs hospitals within networks that did not. We chose this procedure as it is a very common operation for Medicare beneficiaries with a presumed lower baseline need for skilled nursing, and it is commonly targeted by bundled payment programs as spending variation is driven by utilization of postacute care rather than complications.^18^

## Methods

This study was deemed exempt from approval and informed consent by the University of Michigan Institutional Review Board due to use of Medicare claims data. The study followed the Strengthening the Reporting of Observational Studies in Epidemiology (STROBE) reporting guideline.

### Data Source and Study Population

We used the 100% Medicare Provider Analysis and Review (MedPAR) files for January 1, 2016, to December 31, 2017, for nonfederal acute care hospitals. We selected patients undergoing total hip replacement with an inpatient admission, which was identified using the procedure coding system of *International Statistical Classification of Diseases, Tenth Revision, Clinical Modification* (*ICD-10-CM*), from the MedPAR file, with confirmatory *Current Procedural Terminology* codes from the Medicare Carrier File, to define the cohort. We further narrowed the cohort to admissions under the diagnosis-related groups for hip replacements (469 and 470). We included patients aged 66 to 99 years with continuous coverage by fee-for-service Medicare for 3 months before and 6 months after the surgical procedure of interest and excluded nonelective admissions and patients in Medicare Advantage. Comorbidities were identified using *ICD-10-CM* diagnosis codes and defined using the Elixhauser method.^19^ Medicare beneficiary race and ethnicity data (categorized as Black, White, or other [including American Indian or Alaska Native, Asian, Hispanic, and other race or ethnicity]) were captured through Social Security data with categories designated by the Office of Management and Budget to present a complete picture of the patient population. We linked hospitals to the American Hospital Association (AHA) Annual Survey for 2017 to identify our primary exposure variable, vertical integration with an SNF. Vertical integration of SNFs was defined as hospitals and their subsidiaries owning an SNF or the health care system owning an SNF as identified in the AHA survey. Hospitals without vertical SNF integration included hospitals with network affiliation without ownership of SNFs. We also used the AHA survey to abstract hospital characteristics, including bed size, urban designation, nonprofit status, and teaching status as well as network identifiers. Systems, as defined by the AHA, include both multihospital systems and well-diversified single-hospital systems, but we excluded the latter by dropping systems with fewer than 2 hospitals performing hip replacements who did not own an SNF. We excluded hospitals that performed less than 10 eligible hip replacements a year and those with missing information regarding their health care network status.

### Outcomes

Our primary outcomes were postoperative utilization of SNFs and 30-day readmissions. Utilization of SNFs was defined as admission to an SNF within 30 days from the discharge day of the index hospitalization. Thirty-day readmissions were defined as any readmission 30 days after the discharge day of the index hospitalization. Our secondary outcomes were 30-day episode payments, which were abstracted from the MedPAR, carrier, outpatient, and home health agency files. These were classified into total episode payment, index hospitalization, physician, readmission, postacute care, and SNF payments. We used price-standardization methods^20^ previously described^21,22^ to adjust for intended differences in Medicare payment rates by year, wage index, and graduate medical education expenses. Patient level episode payments were winsorized at the 1st and 99th percentiles after price standardization. Winsorization recodes extreme outliers to less extreme values, thereby improving model fit while preserving the underlying signal and without deleting observations.^23^

### Statistical Analysis

Data were analyzed from February 2 to August 8, 2022. We used hierarchical multivariable logistical regression models, accounting for hospital-level random effects, to evaluate dichotomous outcomes with vertical integration of SNFs as a binary variable. We used hierarchical multivariable linear regression accounting for hospital-level random effects to model payments. All models included the following covariates: age, race and ethnicity, sex, Elixhauser comorbidities, and hospital characteristics including size, teaching status, urban designation, and profit status. In addition, models included network factors such as the number of hospitals in the network. We calculated hospital and network volumes by calculating the number of procedures performed by each hospital and network per year after all exclusion criteria were met in our data set. Hospital volume was further stratified into 4 quartiles (highest, above average, below average, and lowest volumes) and included as a candidate covariate. To further characterize the association of hospital volume on utilization of SNFs, an interaction term between hospital volume and vertical integration of SNFs was also included as a candidate covariate.

We also performed a sensitivity analysis for patients admitted to an SNF using hierarchical multivariable logistical and linear regression models with hospital-level random effects, as those patients may be the most likely to be affected by vertical integration of SNFs in a hospital network. Given that SNFs are paid a per diem rate, we specifically looked at length of stay between the 2 groups to determine the pattern behind SNF length of stay on payments in hospital networks with vertical SNF integration. We calculated SNF length of stay for the first admission to an SNF and right-censored the length of stay at 30 days. The SNF length of stay was then categorized into 5- or 10-day increments, and an interaction term between SNF length of stay and vertical integration of SNFs was included in our model for 30-day readmissions.

Statistical analyses were performed using Stata, version 17 (StataCorp LLC). In addition to odds ratios (ORs), cluster-adjusted mean percentages and differences were estimated via Stata’s postestimation command margins, immediately after fitting clustered logistic and linear regression models. This was performed by holding all covariates at the mean and calculating estimated probabilities of the outcome of interest conditional on those covariates being fixed. Two-sided *P* < .05 indicated statistical significance.

## Results

### Cohort Characteristics

We identified 150 788 patients with fee-for-service Medicare coverage who underwent elective total hip arthroplasty in 2016 and 2017 at 1249 hospitals within 305 hospital networks. The selection and exclusion criteria for our cohort are provided in a study flow diagram in eFigure 1 in Supplement 1. The mean (SD) age was 74.3 (6.4) years, 92 550 patients (61.4%) were women and 52 238 (36.8%) were men, and 89 804 (59.6%) had 2 or more coded comorbidities. In terms of race and ethnicity, 7114 patients (4.7%) were Black, 138 172 (91.6%) were White, and 5502 (3.6%) were identified as other race or ethnicity (including Asian, Hispanic, and Native American). A total of 112 082 patients (74.3%) were discharged home, and 33 688 (22.3%) utilized SNFs. There were 8864 patients (5.9%) readmitted within 30 days. Hospitals in a network with vertical integration with SNFs had higher rates of SNF utilization (23.3% vs 21.6%; *P* < .001) and lower 30-day readmissions (5.7% vs 6.0%; *P* = .002). Differences between unadjusted 30-day total payments between the 2 groups were not clinically significant. Demographic data and bivariate comparisons by vertical integration of SNFs are provided in Table 1, and bivariate comparisons of the medical comorbidities and average payments can be seen in eTable 1 in Supplement 1.

**Table 1.** Unadjusted Patient-Level Baseline Characteristics and Utilization Stratified by Vertical Integrations of SNFs Within Hospital Networks

Characteristic	Patient group^a^			*P* value
	All (N = 150 788)	SNF vertical integration		
		No (n = 85 771)	Yes (n = 65 017)	
Age, mean (SD), y	74.3 (6.4)	74.2 (6.4)	74.4 (6.5)	<.001
Sex				
Men	58 238 (38.6)	33 447 (39.0)	24 791 (38.1)	<.001
Women	92 550 (61.4)	52 324 (61.0)	40 226 (61.9)	
Race and ethnicity				
Black	7114 (4.7)	3757 (4.4)	3357 (5.2)	<.001
White	138 172 (91.6)	78 842 (91.9)	59 330 (91.3)	<.001
Other^b^	5502 (3.6)	3172 (3.7)	2330 (3.6)	
Elixhauser comorbidities				
0	19 988 (13.3)	11 460 (13.4)	8528 (13.1)	.003
1	40 996 (27.2)	23 032 (26.9)	17 964 (27.6)	
≥2	89 804 (59.6)	51 279 (59.8)	38 525 (59.3)	
Hospital profit status				
For-profit	11 621 (7.7)	9945 (11.6)	1676 (2.6)	<.001
Not-for-profit	131 685 (87.3)	71 985 (83.9)	59 700 (91.8)	
Other	7482 (5.0)	3841 (4.5)	3641 (5.6)	
Hospital size, No. of beds				
<200	39 768 (26.4)	26 946 (31.4)	12 822 (19.7)	<.001
200-349	38 772 (25.7)	22 109 (25.8)	16 663 (25.6)	
350-499	28 163 (18.7)	16 263 (19.0)	11 900 (18.3)	
≥500	44 085 (29.2)	20 453 (23.8)	23 632 (36.3)	
Teaching hospital	121 369 (80.5)	68 021 (79.3)	53 348 (82.1)	<.001
Urban hospital	141 694 (94.0)	79 239 (92.4)	62 455 (96.1)	<.001
Hospital length of stay, d				
≥2	44 168 (29.3)	24 903 (29.0)	19 265 (29.6)	.047
3-4	94 135 (62.4)	53 732 (62.7)	40 403 (62.1)	
5-6	9557 (6.3)	5492 (6.4)	4065 (6.3)	
≥7	2928 (1.9)	1644 (1.9)	1284 (2.0)	
Discharged home	112 082 (74.3)	63 718 (74.3)	48 364 (74.4)	.67
Discharged to SNF	33 688 (22.3)	18 552 (21.6)	15 136 (23.3)	<.001
SNF length of stay, d^c^				
1-5	2298 (6.8)	1288 (6.9)	1010 (6.7)	<.001
6-10	8977 (26.6)	4547 (24.5)	4430 (29.3)	
11-20	14 025 (41.6)	7846 (42.3)	6179 (40.8)	
≥21	8388 (24.9)	4871 (26.3)	3517 (23.2)	
Hospitals in network, median (IQR)	8 (4-22)	9 (4-33)	7 (4-15)	<.001
Yearly hip replacement operations per hospital, median (IQR)	102 (55-175)	92 (51-162)	115 (61-199)	<.001
Yearly hip replacement operations per network, median (IQR)	508 (249-988)	497 (242-1449)	527 (262-797)	<.001
30-d Readmissions	8864 (5.9)	5179 (6.0)	3685 (5.7)	.002
30-d Mortality	341 (0.2)	193 (0.2)	148 (0.2)	.92
Total mean (SD) payment, $	20 252 (6791)	20 302 (6990)	20 186 (6520)	.001

Abbreviation: SNF, skilled nursing facility.

^a^ Unless otherwise indicated, data are presented as No. (%) of patients. Percentages have been rounded and may not total 100.

^b^ Includes American Indian or Alaska Native, Asian, Hispanic, and other race or ethnicity.

^c^ Includes only patients admitted to SNFs.

### SNF Utilization and Outcomes

**Figure 1.**
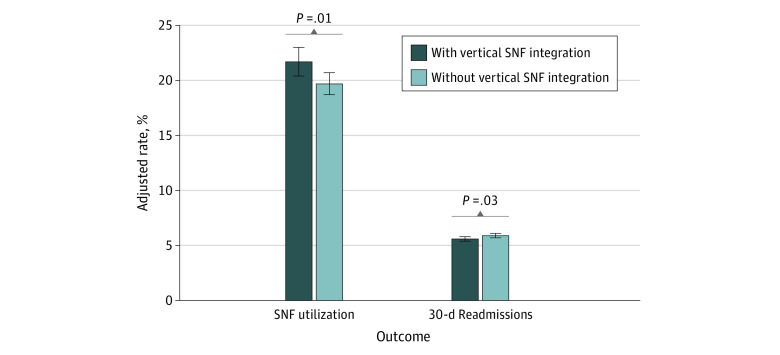
Adjusted Rates of Skilled Nursing Facility (SNF) Utilization and 30-Day Readmissions Stratified by Vertical Integration of SNFs Within Hospital Network Rates are adjusted for medical comorbidities, patient and hospital factors, hospital volume, and network size. Error bars indicate 95% CIs.

After controlling for medical comorbidities, patient and hospital factors, hospital volume, and network size, SNF vertical integration was associated with SNF utilization (21.7% [95% CI, 20.4%-23.0%] vs 19.7% [95% CI, 18.7%-20.7%]; adjusted OR [aOR], 1.15 [95% CI, 1.03-1.29]; *P* = .01). Higher quartiles of hospital surgical volume were associated with decreasing rates of SNF utilization. Table 2 demonstrates the interaction of vertical integration of SNFs and hospital volume as vertically integrated networks utilized SNFs at higher adjusted rates and increasing surgical volume was associated with lower rates of utilization. There was also a significant decrease in 30-day readmissions for patients cared for in hospital networks with SNF vertical integration (5.6% [95% CI, 5.4%-5.8%] vs 5.9% [95% CI, 5.7%-6.1%]; aOR, 0.94 [95% CI, 0.89-0.99]; *P* = .03), and displayed graphically in Figure 1. Thirty-day episode payments were slightly lower for hospital networks with SNF vertical integration ($20 230 [95% CI, $20 035-$20 425] vs $20 487 [95% CI, $20 314-$20 660]; difference, −$275 [95% CI, −$15 to −$498]; *P* = .04), and there were no significant differences in any individual payment component.

**Table 2.** Adjusted Outcomes and Payments Stratified by Vertical Integration of SNFs Within Hospital Networks^a^

Outcome	Adjusted OR or payment difference (95% CI)	*P* value	Adjusted rate or payment (95% CI)	
			No SNF vertical integration (n = 85 771)	SNF vertical integration (n = 65 017)
SNF admission, OR (95% CI)	1.15 (1.03 to 1.29)	.01	19.7 (18.7 to 20.7)	21.7 (20.4 to 23.0)
SNF admission by hospital volume, OR (95% CI)				
Lowest	1 [Reference]	<.001	25.0 (23.6 to 26.3)	27.7 (26.1 to 29.4)
Below average	0.83 (0.74 to 0.95)		23.0 (21.6 to 24.4)	25.0 (23.3 to 26.7)
Above average	0.65 (0.56 to 0.77)		22.0 (20.3 to 23.7)	21.5 (19.7 to 23.3)
Highest	0.60 (0.49 to 0.74)		18.1 (15.7 to 20.5)	20.3 (18.1 to 22.6)
30-d readmission, OR (95% CI)	0.94 (0.89 to 0.99)	.03	5.9 (5.7 to 6.1)	5.6 (5.4 to 5.8)
30-d readmission by SNF LOS, OR (95% CI)				
None	1 [Reference]	NA	3.8 (3.6 to 3.9)	3.3 (3.1 to 3.5)
1-5 d	19.81 (17.52 to 22.41)	<.001	41.8 (39.0 to 44.6)	40.9 (37.8 to 44.0)
6-10 d	4.63 (4.21 to 5.08)	<.001	15.0 (13.9 to 16.1)	12.2 (11.2 to 13.3)
11-20 d	3.43 (3.16 to 3.72)	<.001	11.6 (10.9 to 12.4)	10.9 (10.1 to 11.8)
≥21 d	1.30 (1.15 to 1.48)	<.001	4.8 (4.3 to 5.4)	4.8 (4.1 to 5.4)
Payment difference (95% CI), $				
Mean total 30-d	−257 (−15 to −498)	.04	20 487 (20 314 to 20 660)	20 230 (20 035 to 20 425)
Index hospitalization	−39 (−48 to 5)	NA	13 729 (13 607 to 13 833)	13 690 (13 592 to 13 806)
Physician services	−15 (−21 to 40)	NA	2403 (2281 to 2530)	2388 (2275 to 2419)
Mean postacute care	−202 (−383 to 30)	NA	4354 (4208 to 4463)	4152 (4080 to 4254)
Mean readmission	−24 (−63 to 17)	NA	566 (544 to 597)	542 (516 to 578)
Readmission if present	−10 (−374 to 350)	NA	11 981 (11 754 to 12 186)	11 971 (11 699 to 12 246)

Abbreviations: LOS, length of stay; NA, not applicable; OR, odds ratio; SNF, skilled nursing facility.

^a^ Odds ratios and rates are adjusted for medical comorbidities, patient and hospital factors, hospital volume, and network size. Payments are price standardized, winsorized, and adjusted for medical comorbidities, patient and hospital factors, hospital volume, and network size.

### Sensitivity Analysis

Compared with patients who did not utilize an SNF, patients who utilized an SNF tended to be older (mean [SD] age, 73.4 [5.9] years vs 77.5 [7.1] years, respectively; *P* < .001) and to have more comorbidities (65 268 [55.7%] vs 24 536 [72.8%], respectively, had ≥2 Elixhauser comorbidities; *P* < .001). The results of bivariate comparisons of demographics, hospital characteristics, and payments for patients admitted to an SNF by vertical integration of SNFs can be seen in the eTable 2 in Supplement 1. A bivariate analysis of SNF length of stay is seen in Table 1 and demonstrates that networks without vertical integration of SNFs have longer length of stays and are more likely to have lengths of stay of 30 days or greater, which is displayed as a histogram in eFigure 2 in Supplement 1. For this subgroup analysis, no significant difference in readmissions was found for patients who underwent surgery in hospital networks with SNF vertical integration vs those who did not (13.8% [95% CI, 13.0%-14.5%] vs 14.7% [95% CI, 14.3%-15.8%]; aOR, 0.93 [95% CI, 0.85-1.01]; *P* = .08) after controlling for medical comorbidities, patient and hospital factors, hospital volume, and network size. Total 30-day payments were modestly but significantly lower (−$507 [95% CI, −$215 to −$754]; *P* < .001) for patients in networks with vertical SNF integration. Payments to SNFs (−$389 [95% CI, −$211 to −$568]; *P* < .001) and total post–acute care payments (−$452 [95% CI, −$232 to −$606]; *P* < .001) were both decreased for hospital networks with vertical integration of SNFs (Table 3).

**Table 3.** Adjusted Outcomes and Payments for Patients Admitted to an SNF Stratified by Vertical Integration of SNFs Within Hospital Networks^a^

Outcomes	Adjusted OR or payment difference	*P* value	Adjusted rate or payment (95% CI)	
			No SNF vertical integration (n = 18 552)	SNF vertical integration (n = 15 136)
30-d Readmission, OR (95% CI)	0.93 (0.85 to 1.01)	.08	14.7 (14.3-15.8)	13.8 (13.0-14.5)
30-d Readmissions by SNF LOS, OR (95% CI)				
1-5 d	1 [Reference]	NA	47.3 (44.3 to 50.3)	46.3 (42.9 to 409.7)
6-10 d	0.23 (0.20 to 0.27)	<.001	17.9 (16.5 to 19.2)	14.6 (13.3 to 15.8)
11-20 d	0.17 (0.15 to 0.20)	<.001	14.0 (13.1 to 14.9)	13.1 (12.0 to 14.1)
≥21 d	0.06 (0.05 to 0.08)	<.001	5.9 (5.2 to 6.6)	5.8 (5.0 to 6.6)
Payments, difference (95% CI), $				
Mean total 30-d	−507 (−215 to −754)	<.001	27 067 (26 885 to 27 254)	26 583 (26 356 to 26 769)
Index hospitalization	−51 (−97 to 4)	NA	14 250 (14 183 to 14 294)	14 206 (14 166 to 14 257)
Physician services	4 (−21 to 39)	NA	3154 (3127 to 3192)	3153 (3055 to 3228)
Mean postacute	−452 (−232 to −606)	<.001	9658 (9607 to 9778)	9205 (8958 to 9347)
Mean readmission	−7 (−151 to 82)	NA	1301 (1223 to 1379)	1266 (1179 to 1354)
Readmission if present	237 (−244 to 723)	NA	12 969 (12 655 to 13 284)	13 209 (12 852 to 13 566)

Abbreviations: LOS, length of stay; NA, not applicable; OR, odds ratio; SNF, skilled nursing facility.

^a^ Odds ratios and rates are adjusted for medical comorbidities, patient and hospital factors, hospital volume, and network size. Payments are price standardized, winsorized, and adjusted for medical comorbidities, patient and hospital factors, hospital volume, and network size.

**Figure 2.**
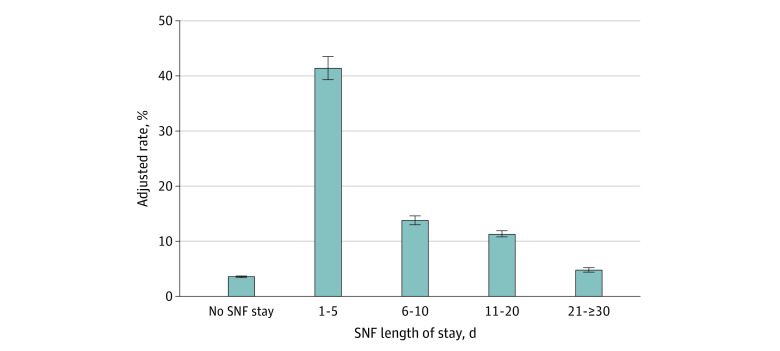
Adjusted 30-Day Readmission Rates Based on Skilled Nursing Facility (SNF) Length of Stay Rates are adjusted for medical comorbidities, patient and hospital factors, hospital volume, and network size. Error bars indicate 95% CIs.

We also performed a sensitivity analysis of factors associated with 30-day readmissions. For this analysis, adjusted readmission rates were particularly low for patients who were not sent to an SNF at 3.6% (95% CI, 3.4%-3.7%; *P* < .001) after controlling for medical comorbidities, patient and hospital factors, hospital volume, and network size. Adjusted 30-day readmission rates were significantly higher for patients with an SNF length of stay less than 5 days at 41.3% (95% CI, 39.2%-43.3%; *P* < .001), which decreased to 13.8% (95% CI, 13.0%-14.5%; *P* < .001) for those with a 6- to 10-day SNF stay, and further decreased over the remaining intervals (Figure 2). There was no statistically significant interaction between vertical integration of SNFs and SNF length of stay, which is demonstrated quantitatively in in Table 2. Similar trends were seen for the subgroup of patients who were admitted to an SNF (Table 3).

## Discussion

The present cross-sectional study evaluating the association of SNF integration within hospital networks with outcomes and payments among Medicare beneficiaries had 3 principal findings. First, we found that hospital networks integrated with an SNF were more likely to utilize SNFs for patients undergoing hip replacement. Second, we found that patients undergoing surgery at hospitals in a network integrated with an SNF had overall lower readmissions rates. Finally, despite higher utilization of SNFs among hospital networks that are integrated with SNFs, total episode payments were slightly lower due to lower readmission rates and shorter SNF lengths of stay. Taken together, these findings suggest that vertical integration of SNFs in hospital networks did not adversely affect costs or outcomes as we initially hypothesized.

Our study found that health care networks with vertical integration of SNFs utilized SNFs at modestly higher rates, which extends prior work that found that hospital ownership of an SNF provided a 9% absolute contribution to the adjusted SNF utilization rate of 51% for patients undergoing emergency surgery.^17^ Considering current trends to transform total hip arthroplasty into an outpatient procedure,^24^ our findings could possibly be interpreted as overutilization. The current diagnosis-related group–based reimbursement system, which reimburses hospitals a fixed amount per procedure regardless of length of stay, incentivizes hospitals to limit length of stay and could result in patients being discharged to post–acute care settings such as SNFs to open hospitals beds and increase efficiency. Vertical integration would allow hospital networks to increase throughput while still capturing the revenue from downstream phases of health care delivery. This interpretation would be further supported by our findings that SNF lengths of stay in vertically integrated health care networks tend to be shorter.

Readmission rates were lower for hospital networks with vertical integrations of SNFs. Prior studies that have focused on SNFs with direct hospital ownership^13,15,16^ or referral linkage^25,26^ have found similar trends in readmission rates. Taken together, these findings suggest that hospital networks could be leveraging vertical integration with post–acute health care services to improve care coordination to reduce readmissions,^15,27^ although other studies have not replicated those findings.^13^ Successful mechanisms were elucidated in a mixed-methods study that found hospital networks with preferred networks of SNFs lowered readmissions by leveraging existing relationships with post–acute care facilities, managing data effectively, and understanding drivers of cost of care beyond publicly available quality metrics.^10^ Quantifying the degree of integration is impossible with currently available data but would be helpful to ameliorate concerns that hospital networks that vertically integrate with SNFs are already more conscientious about quality and costs. Our analysis of the association of SNF length of stay with 30-day readmission rates identified significantly higher adjusted readmission rates for patients within the first 5 days of an SNF stay, regardless of vertical integration. These results could support funding initiatives to improve care coordination early in an SNF stay by hospital networks or justify early postoperative follow-up appointments for patients with postsurgical stays in SNFs.

In our study, vertical integration of SNFs was associated with modest decreases in total 30-day payments for Medicare beneficiaries undergoing elective hip replacement. Chhabra et al^28^ first examined surgical payments for hip replacements associated with health care networks and demonstrated wide variation in payments driven by post–acute care utilization. While there are concerns that hospitals with vertically integrated SNFs steer more profitable patients toward their own SNFs,^29^ studies of medical cohorts found significant cost savings for Medicare overall when SNFs were hospital-based^13^ or vertically integrated with a hospital.^15^ Our work extends these findings to patients undergoing surgery, as we found lower post–acute care payments for hospital networks with vertical SNF integration were driven by overall lower readmission rates and shorter SNF lengths of stay. While the goal would be to understand the appropriateness of SNF use for our exposure, this is difficult to assess with claims data. Nonetheless, these findings could be used to alleviate worries that vertical integration leads to overutilization of postacute care and higher payments to the hospital networks from inappropriate incentives.^9^

As both public and private payers expand the uptake of bundled payment initiatives,^30,31^ hospitals are incentivized to decrease utilization of potentially unwarranted services. However, this may be blunted with vertically integrating these services, as hospitals may recoup reimbursement from SNFs with overutilization by fee-for-service patients despite potential penalties from the bundled payment program. The results of our study may temper concerns that increasing vertical integration of postacute care would lead to rising health care costs. We found that vertical integration of SNFs was associated with lower overall payments, despite higher rates of SNF utilization due to shorter SNF lengths of stay. Understanding the appropriateness of these short SNF stays will be necessary to understand whether this is in fact overutilization. Patient expectations and health care practice patterns play a prominent role in SNF utilization rates for lower-risk procedures,^28^ as a recent quality improvement initiative focusing on changing case management and patient expectations successfully lowered rates of SNF utilization for this patient population.^32^ With the current level of attention paid to hospital consolidations, policy makers could consider alternative payment models at the hospital network level to further align incentives with postacute care. Piloting a hospital network-level alternative payment model for joint replacements is particularly attractive as spending variation is driven by post–acute care utilization and should be more easily modifiable^32^ than improving complications rates that drive spending for other common procedures such as colectomy.^33^ A bundled payment program aimed at the hospital network level could encourage care coordination within the network while incentivizing decreased utilization of potentially unnecessary services and controlling overall costs.

### Limitations

This study should be interpreted in the context of several limitations. This is a retrospective study using administrative claims data and relies on accurate coding of diagnosis, comorbidities, and complications. There are limitations in clinical details that can result in confounding from unmeasured factors such as sociodemographic factors, uncoded comorbidity, and patient severity. The population studied is older than 65 years, and this may limit the generalizability of our findings. We were unable to measure the degree of clinical and financial integration for the hospitals within each health care network or with the SNFs. Because data on SNF ownership is limited, we were unable to determine whether patients were sent to the SNF that the hospital network owned. Furthermore, the ability to quantify or understand the quality of nursing care delivered within the SNFs was limited and may have an interaction with the results seen in our study based on prior research.^13,34^ We did not include any geographic data in our analysis, and prior studies have demonstrated that geography plays a crucial role in SNF utilization.^35^ Our design is cross-sectional rather than longitudinal due to limitations in the existing data, as the AHA Annual Survey system identifiers do not stably identify merged hospitals over time.

## Conclusions

As hospital networks continue to merge, there is an opportunity to achieve higher-value health care through improved integration with postacute care. Our cross-sectional study demonstrated that vertical integration of SNFs in a hospital network was associated with higher rates of SNF utilization and lower readmission rates among Medicare beneficiaries undergoing elective total hip replacements with lower overall payments. For patients requiring an SNF, 30-day episode payments were lower as hospital networks with vertical integration of SNFs were associated with shorter SNF length of stays. Thirty-day readmission rates were highest for patients with an SNF length of stay less than 5 days. These findings support the purported value of integrating SNFs into hospital networks but also suggest there is room for improving the care of patients requiring postoperative SNF care early in their stay.

## Notes

Supplement 1. **eFigure 1.** CONSORT Diagram of the Study Population **eTable 1.** Unadjusted Patient-Level Medical Comorbidities by Vertical Integrations of Skilled Nursing Facility Within Hospital Networks **eTable 2.** Unadjusted Patient-Level Characteristics, Utilization, and Payments Stratified by Vertical Integrations of SNF Within Hospital Networks for Patients Admitted to an SNF **eFigure 2.** Histogram of Skilled Nursing Facility Length of Stay for Patients Admitted to a Skilled Nursing Facility by Vertical Integration With Skilled Nursing Facilities

Supplement 2.
Data Sharing Statement
