# Supplementary material for: Association of Skilled Nursing Facility Ownership by Health Care Networks With Utilization and Spending
Source: JAMA Netw Open. 2023 Feb 20;6(2):e230140. doi: 10.1001/jamanetworkopen.2023.0140 (PMC9941887; doi:10.1001/jamanetworkopen.2023.0140)
Supplement: Supplement 1. — eFigure 1. CONSORT Diagram of the Study Population eTable 1. Unadjusted Patient-Level Medical Comorbidities by Vertical Integrations of Skilled Nursing Facility Within Hospital Networks eTable 2. Unadjusted Patient-Level Characteristics, Utilization, and Payments Stratified by Vertical Integrations of SNF Within Hospital Networks for Patients Admitted to an SNF eFigure 2. Histogram of Skilled Nursing Facility Length of Stay for Patients Admitted to a Skilled Nursing Facility by Vertical Integration With Skilled Nursing Facilities [file jamanetwopen-e230140-s001.pdf]

Supplementary Online Content

Kalata S, Howard R, Diaz A, Nuliyahu U, Ibrahim AM, Nathan H. Association of skilled nursing facility ownership by health care networks with utilization and spending. *JAMA Netw Open*. 2023;6(2):e230140. doi:10.1001/jamanetworkopen.2023.0140

eFigure 1. CONSORT Diagram of the Study Population
eTable 1. Unadjusted Patient-Level Medical Comorbidities by Vertical Integrations of Skilled Nursing Facility Within Hospital Networks
eTable 2. Unadjusted Patient-Level Characteristics, Utilization, and Payments Stratified by Vertical Integrations of SNF Within Hospital Networks for Patients Admitted to an SNF
eFigure 2. Histogram of Skilled Nursing Facility Length of Stay for Patients Admitted to a Skilled Nursing Facility by Vertical Integration With Skilled Nursing Facilities

This supplementary material has been provided by the authors to give readers additional information about their work.

© 2023 Kalata S et al. *JAMA Network Open*.

eFigure 1. CONSORT Diagram of the Study Population

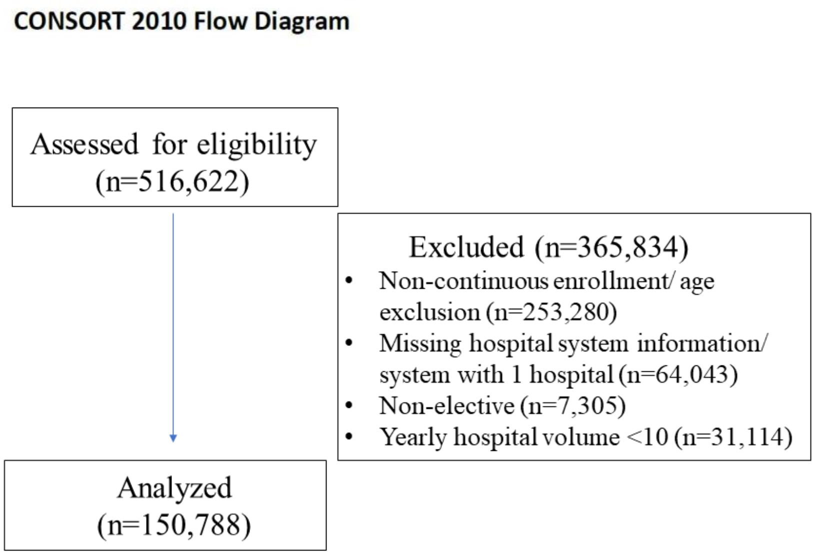

eTable 1. Unadjusted Patient-Level Medical Comorbidities by Vertical Integrations of Skilled Nursing Facility Within Hospital Networks

© 2023 Kalata S et al. *JAMA Network Open*.

	All Patients (n=150,788)		No SNF Vertical Integration (n=85,771)		SNF Vertical Integration (n=65,017)		p
Obesity	27,560	18.3%	15,547	18.1%	12,013	18.5%	0.081
Hypertension	104,341	69.2%	59,326	69.2%	45,015	69.2%	0.78
Diabetes	26,158	17.3%	15,022	17.5%	11,136	17.1%	0.050
Congestive Heart Failure	6,141	4.1%	3,428	4.0%	2,713	4.2%	0.087
Major Vascular Disease	8,294	5.5%	4,560	5.3%	3,734	5.7%	<0.001
Pulmonary Hypertension	221	0.1%	128	0.1%	93	0.1%	0.76
Peripheral Arterial Disease	5,011	3.3%	2,864	3.3%	2,147	3.3%	0.69
COPD	22,754	15.1%	13,149	15.3%	9,605	14.8%	0.003
Renal Failure	12,025	8.0%	6,869	8.0%	5,156	7.9%	0.58
Peptic Ulcers	522	0.3%	313	0.4%	209	0.3%	0.15
Liver Disease	1,545	1.0%	869	1.0%	676	1.0%	0.61
Solid Organ Cancer	1,134	0.8%	619	0.7%	515	0.8%	0.12
Cancer with Metastasis	139	0.1%	77	0.1%	62	0.1%	0.72
Weight Loss	773	0.5%	461	0.5%	312	0.5%	0.12
Fluid/Electrolyte Disorders	11,981	7.9%	6,645	7.7%	5,336	8.2%	0.001
Blood Loss Anemia	1,147	0.8%	589	0.7%	558	0.9%	<0.001
Anemia due to Deficiency	13,282	8.8%	7,824	9.1%	5,458	8.4%	<0.001
Coagulopathy	3,323	2.2%	1,862	2.2%	1,461	2.2%	0.32
Lymphoma	553	0.4%	297	0.3%	256	0.4%	0.13
Hypothyroidism	29,060	19.3%	16,534	19.3%	12,526	19.3%	0.96
Collagen/Rheum Disorders	6,695	4.4%	3,819	4.5%	2,876	4.4%	0.79
Neurologic Disease	7,560	5.0%	4,333	5.1%	3,227	5.0%	0.44
Paralysis	583	0.4%	317	0.4%	266	0.4%	0.22
Depression	18,218	12.1%	10,477	12.2%	7,741	11.9%	0.068
Psychiatric Disorders	1,347	0.9%	754	0.9%	593	0.9%	0.50
Alcohol abuse	1,294	0.9%	716	0.8%	578	0.9%	0.26
Drug abuse	603	0.4%	321	0.4%	282	0.4%	0.070
Mean Index Hospitalization	$13,516		$13,521		$13,509		0.052
Mean Physician Payment	$2,192		$2,185		$2,201		<0.001
Mean Post-acute Payment	$3,918		$3,946		$3,882		0.009
Mean Readmission Payment	$553		$563		$541		0.14
Mean SNF Payment	$1,785		$1,785		$1,784		0.96
Mean 30-day Total Payment	$20,252		$20,302		$20,187		0.001

SNF=Skilled nursing facility
COPD=Chronic Obstructive Pulmonary Disease

© 2023 Kalata S et al. *JAMA Network Open*.

eTable 2. Unadjusted Patient-Level Characteristics, Utilization, and Payments Stratified by Vertical Integrations of SNF Within Hospital Networks for Patients Admitted to an SNF

	All Patients (n=33,688)		No SNF Vertical Integration (n=18,552)		SNF Vertical Integration (n=15,136)		p
Age (SD)	77.5 (7.1)		77.5 (7.0)		77.6 (7.1)		0.069
Male	9,351	27.8%	5,156	27.8%	4,195	27.7%	0.88
Female	24,337	72.2%	13,396	72.8%	10,941	72.3%	
Black	2,234	6.7%	1,165	6.3%	1,069	7.1%	0.004
White	30,511	91.3%	16,845	91.6%	13,666	91.0%	0.063
Obesity	7,079	21.0%	3,828	20.6%	3,251	21.5%	0.058
Hypertension	25,540	75.8%	14,054	75.8%	11,486	75.9%	0.78
Diabetes	7,464	22.2%	4,134	22.3%	3,330	22.0%	0.53
Congestive Heart Failure	2,461	7.3%	1,347	7.3%	1,114	7.4%	0.73
Major Vascular Disease	2,548	7.6%	1,346	7.3%	1,202	7.9%	0.018
Pulmonary Hypertension	92	0.3%	53	0.3%	39	0.3%	0.62
Peripheral Arterial Disease	1,630	4.8%	905	4.9%	725	4.8%	0.71
COPD	6,699	19.9%	3,749	20.2%	2,950	19.5%	0.10
Renal Failure	4,098	12.2%	2,242	12.1%	1,856	12.3%	0.62
Peptic Ulcers	169	0.5%	100	0.5%	69	0.5%	0.28
Liver Disease	401	1.2%	217	1.2%	184	1.2%	0.70
Solid Organ Cancer	286	0.8%	148	0.8%	138	0.9%	0.26
Cancer with Metastasis	42	0.1%	24	0.1%	18	0.1%	0.79
Weight Loss	331	1.0%	200	1.1%	131	0.9%	0.049
Fluid/Electrolyte Disorders	4,262	12.7%	2,291	12.3%	1,971	13.0%	0.065
Blood Loss Anemia	426	1.3%	213	1.1%	213	1.4%	0.034
Anemia due to Deficiency	4,241	12.6%	2,403	13.0%	1,838	12.1%	0.026
Coagulopathy	1,091	3.2%	585	3.2%	506	3.3%	0.33
Lymphoma	159	0.5%	84	0.5%	75	0.5%	0.57
Hypothyroidism	7,751	23.0%	4,273	23.0%	3,478	23.0%	0.91
Collagen/Rheum Disorders	1,835	5.4%	1,010	5.4%	825	5.5%	0.98
Neurologic Disease	2,732	8.1%	1,539	8.3%	1,193	7.9%	0.17
Paralysis	267	0.8%	141	0.8%	126	0.8%	0.46
Depression	5,360	15.9%	2,967	16.0%	2,393	15.8%	0.65
Psychiatric Disorders	569	1.7%	305	1.6%	264	1.7%	0.48
Alcohol abuse	334	1.0%	190	1.0%	144	1.0%	0.50
Drug abuse	212	0.6%	108	0.6%	104	0.7%	0.23
Elixhauser Comorbidities							0.003
0	2,416	7.2%	1,398	7.5%	1,018	6.7%	
1	6,736	20.0%	3,629	19.6%	3,107	20.5%	
2 or more	24,536	72.8%	13,525	72.9%	11,011	72.7%	
Hospital Profit Status							<0.001
For-Profit	2,450	7.3%	2,043	11.0%	407	2.7%	
Not-For-Profit	29,534	87.7%	15,657	84.4%	13,877	91.7%	
Other	1,704	5.1%	852	4.6%	852	5.6%	
Hospital Bed Size							<0.001
<200 beds	8,745	26.0%	5,552	29.9%	3,193	21.1%	
200-349 beds	8,960	26.6%	4,942	26.6%	4,018	26.5	
350-499 beds	6,451	19.1%	3,720	20.1%	2,731	18.0%	
>500 beds	9,532	28.3%	4,338	23.4%	5,194	34.3%	
Teaching Hospital	27,214	80.8%	14,915	80.4%	12,299	81.3%	0.046
Urban Hospital	31,759	94.3%	17,264	93.1%	14,495	95.8%	<0.001

© 2023 Kalata S et al. *JAMA Network Open*.

	All Patients (n=33,688)		No SNF Vertical Integration (n=18,552)		SNF Vertical Integration (n=15,136)		p
Hospital Length of Stay							.008
At Least 2 Days	500	1.5%	241	1.3%	259	1.7%	
3-4 Days	26,391	78.3%	14,528	78.3%	11,863	78.4%	
5-6 Days	5,061	15.0%	2,837	15.3%	2,224	14.7%	
7+ Days	1,736	5.2%	946	5.1%	790	5.2%	
Yearly Median (IQR) Surgeries/ Hospital	91(47-160)		85(43-144)		102(51-181)		<0.001
Yearly Median (IQR) Surgeries/Network	514(249-995)		48 (225-1,346)		585(267-853)		0.30
30-Day Readmissions	4,792	14.2%	2,720	14.7%	2,072	13.7%	.011
30-Day Mortality	100	0.3%	56	0.3%	44	0.3%	0.85
Mean Index Hospitalization	$13,752		$13,758		$13,746		0.55
Mean Physician Payment	$2,674		$2,644		$2,711		<0.001
Mean Post-acute Payment	$8,885		$9,044		$8,690		<0.001
Mean Readmission Payment	$1,242		$1,257		$1,224		0.49
Mean SNF Payments	$7,479		$7,659		$7,259		<0.001
Mean 30-day Total Payment	$26,662		$26,831		$26,456		<0.001

SNF=Skilled nursing facility
SD=Standard Deviation
COPD=Chronic Obstructive Pulmonary Disease
IQR=Interquartile Range

© 2023 Kalata S et al. *JAMA Network Open*.

eFigure 2. Histogram of Skilled Nursing Facility Length of Stay for Patients Admitted to a Skilled Nursing Facility by Vertical Integration With Skilled Nursing Facilities

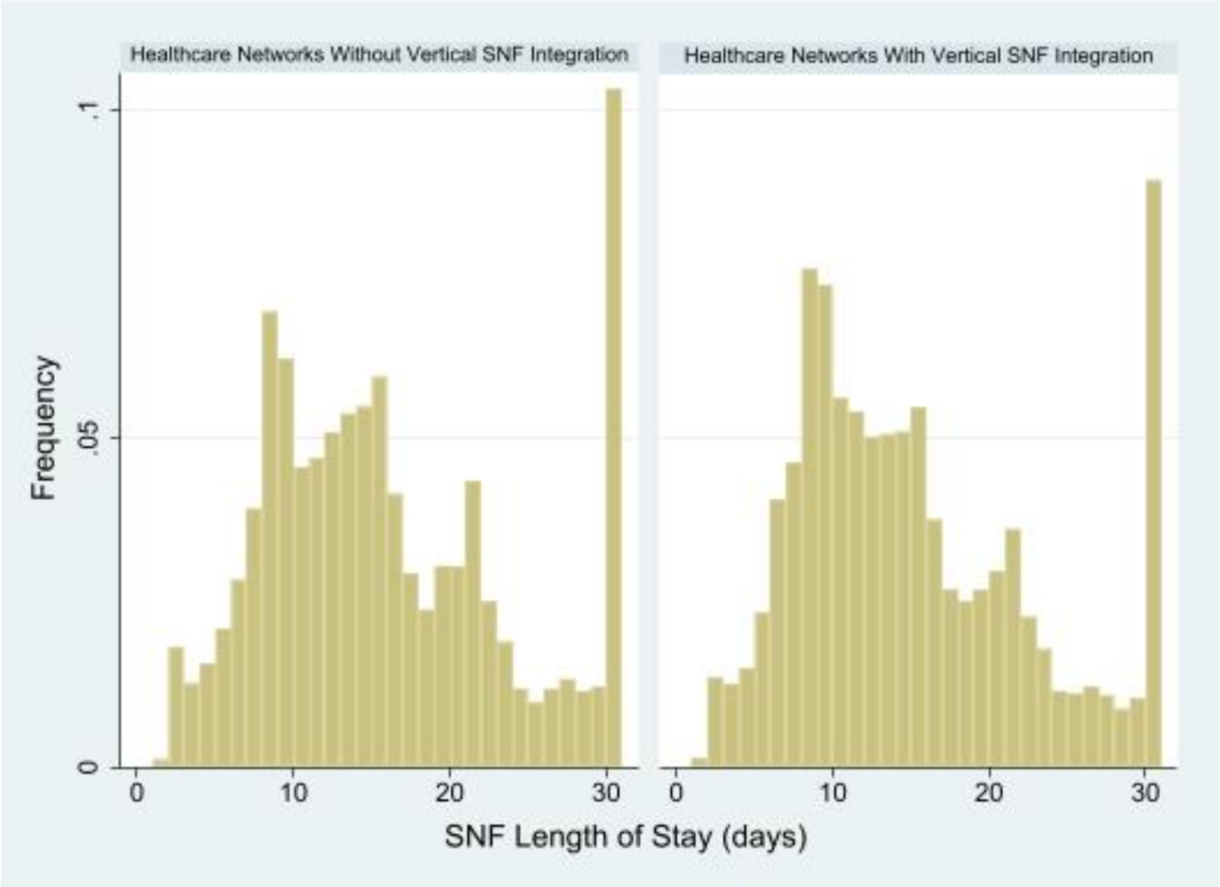

*Skilled nursing facility length of stay was censored at 30 days

© 2023 Kalata S et al. *JAMA Network Open*.
