# Supplementary material for: Association of Skilled Nursing Facility Ownership by Health Care Networks With Utilization and Spending
Source: JAMA Netw Open. 2023 Feb 20;6(2):e230140. doi: 10.1001/jamanetworkopen.2023.0140 (PMC9941887; doi:10.1001/jamanetworkopen.2023.0140)
Supplement: Supplement 2. — Data Sharing Statement [file jamanetwopen-e230140-s002.pdf]

Data Sharing Statement

Kalata. Association of Skilled Nursing Facility Ownership by Health Care Networks With Utilization and Spending. *JAMA Netw Open*. Published February 20, 2023. doi:10.1001/jamanetworkopen.2023.0140

Data
Data available: No

Additional Information
Explanation for why data not available: We utilized 100% Medicare claims data and the American Hospital Association survey data which are obtainable by interested parties.
